# Magnetoencephalography (MEG) Augments Semiology in Stereotactic Electroencephalography (SEEG) Evaluations

**DOI:** 10.3390/brainsci16080778

**Published:** 2026-07-23

**Authors:** Andrew J. Zillgitt, Garnett C. Smith, David E. Burdette, Cesar A. Serrano Almeida, Anant Krishnan, Michael D. Staudt, Paul Ferrari

**Affiliations:** 1 Department of Neurology, Corewell Health William Beaumont University Hospital, Royal Oak, MI 48073, USA; 2 Department of Pediatrics, Division of Pediatric Neurology, Corewell Health William Beaumont University Hospital Florence and Richard McGrien Pediatric Neuroscience Center, Royal Oak, MI 48073, USA; 3 Department of Neurosciences, Corewell Health and Michigan State University College of Human Medicine, Grand Rapids, MI 49503, USA; 4 Department of Neurological Surgery, Corewell Health William Beaumont University Hospital Florence and Richard McGrien Pediatric Neuroscience Center, Royal Oak, MI 48073, USA; 5 Department of Diagnostic Radiology, Corewell Health William Beaumont University Hospital, Royal Oak, MI 48073, USA; 6 Department of Neurological Surgery, University Hospitals Cleveland Medical Center, Cleveland, OH 44106, USA; 7 School of Medicine, Case Western Reserve University, Cleveland, OH 44106, USA; 8 Jack H Miller Magnetoencephalography Center, Helen DeVos Children’s Hospital, Grand Rapids, MI 49503, USA

**Keywords:** magnetoencephalography (MEG), stereotactic electroencephalography (SEEG), semiology, epilepsy surgery

## Abstract

The foundation of a successful stereotactic electroencephalography (SEEG) evaluation has remained relatively unchanged since the first SEEG implantation in 1957 and is predicated on a robust semiology-driven hypothesis. Although epilepsy presurgical evaluation has expanded since the 1950s with the introduction of new technology, e.g., brain magnetic resonance imaging (MRI), few, if any, noninvasive diagnostic studies provide significant complementary information to the epilepsy presurgical evaluation as magnetoencephalography (MEG). As SEEG becomes increasingly ubiquitous as the intracranial EEG modality of choice for more comprehensive epilepsy centers, the importance of MEG in the epilepsy presurgical evaluation also increases. In this review, the use of MEG in addition to seizure semiology in the development of an SEEG implantation strategy will be reviewed and illustrated with two representative cases. Finally, within this review, epilepsy surgical outcomes from presurgical evaluations that incorporated MEG will be discussed.

## 1. Introduction

Many people with epilepsy (PWE) achieve seizure-freedom after beginning treatment with antiseizure medications (ASMs). However, approximately 30–40% of PWE continue to experience seizures despite ASM trials [1,2,3]. In patients with drug-resistant epilepsy (DRE), defined as persistent seizures despite two adequate ASM trials, epilepsy surgery may be the most appropriate treatment [3,4,5,6,7,8]. People with drug-resistant epilepsy must complete a thorough epilepsy presurgical evaluation before proceeding to epilepsy surgery [9]. The foundation of any epilepsy evaluation, including an epilepsy presurgical evaluation, begins with a thorough history and a detailed review of a patient’s seizure semiology [10]. After this, a series of diagnostic studies may be completed, most commonly, inpatient video-EEG monitoring, brain MRI, positron emission tomography (PET), single-photon emission computed tomography (SPECT), and neuropsychology testing [9]. Although MEG has demonstrated impressive results in the epilepsy presurgical evaluation, it has been used in a median of only 5% of presurgical evaluations [9].

## 2. Magnetoencephalography (MEG)

Magnetoencephalography is a noninvasive diagnostic study that directly measures neuronal activity by recording the magnetic flux created from dendritic, intracellular, and electrical currents of neurons [11]. Brain localization of epileptiform activity is accomplished through mathematical modeling of spike-generated magnetic fields within a model of the head and then co-registering those sources with MRI. The resulting image is often called a Magnetic Source Image (MSI). Throughout the remainder of this paper, MEG and MSI may be used interchangeably.

There are several concepts to consider with regard to MEG source localization. Firstly, the inverse problem consists of estimating the location of a neuronal generator or generators given the distribution of magnetic flux patterns across the MEG sensors [12,13]. Because unlimited neuronal source configurations can account for any given MEG sensor pattern, the inverse problem is mathematically ill-posed and does not have a unique solution [12]. However, if certain prior simplifying assumptions are made about the underlying source(s) and the model used, robust source estimation can be accomplished.

The first of these assumptions is the model. The equivalent current dipole (ECD) model is the workhorse of most EEG and MEG source localization methods. In its simplest form, the single ECD model assumes that the neuronal generator of a magnetic field at a single time point can be accurately modeled by a single point source with set parameters of location, current orientation, and current moment or strength.

Secondly, assuming a volume conductor model, also known as a head model, within which the magnetic field travels, the predicted sensor amplitudes can be calculated from any point source in the head. This simulation from model source to sensors is called the forward solution or lead field and always has a unique mathematical solution. In the case of MEG, the magnetic permeability of the various substrates of the head (e.g., brain tissue, bone, and cerebral fluid, etc.) is virtually non-existent and, more importantly, homogeneous throughout the brain. This allows the primary MEG signals from the post-synaptic potentials to travel relatively undistorted through the head, unlike electrocerebral activity measured by EEG. Additionally, in a perfectly spherical conductor, the secondary magnetic fields from return currents cancel out, resulting in a relatively elegant and computationally efficient model [12]. This is why, throughout the tenure of MEG as a localization tool, the single spherical head model has been the standard.

However, the model’s performance for a single sphere breaks down in regions of the brain that are not spherical, like around the temporal and frontal poles [14]. Likewise, in patients with prior resections or brain abnormalities that cause large cavities in the brain, these may also introduce errors when using the single sphere. This is because while the primary MEG fields do not experience distortion, the secondary return currents of the electric fields in these areas introduce secondary magnetic fields that do not cancel out. For this reason, and with the advent of more powerful computational resources, realistic head shape models have been increasingly used in most modern localization software, e.g., boundary element models (BEMs). Dipole modeling in the vicinity of large cavities in the brain is still an ongoing area of development.

The inverse solution using a single ECD involves an iterative search of the head space for the dipole parameters, e.g., location (x, y, z), current orientation, and moment (nAm), which minimize the least square differences between the observed MEG data and the ECD model. There are several statistical parameters that may be used to assess the quality of the ECD [13]. These parameters may include the dipole moment (Q), correlation coefficient (R), goodness of fit (GoF), root mean square (RMS), and confidence volume (CV) [13,15]. Table 1 outlines the details of these ECD statistical parameters. Some of these metrics are redundant and in practice, only a few are typically used for localizing epileptiform activity. The CV defines the 3D spatial region—usually an ellipsoid—around an estimated ECD that statistically encompasses the true, noise-free location of the brain source with a specific probability (typically 95%) [16]. GoF defines how accurately an equivalent current dipole model explains the measured magnetic field data. It is typically expressed as a percentage from 0% to 100%, calculated as one minus the residual variance (RV) between the observed and modeled magnetic fields [17]. In general, the dipole model of an epileptiform discharge should have a GoF ≥ 80%, CV less than 1 cm^3^, and a Q between 100 and 500 nAm [13]. Supplementary Material Figures S2–S4 outline the steps in the clinical evaluation of interictal epileptiform activity using the ECD on simultaneous EEG-MEG and are based on the American Clinical Magnetoencephalography Society (ACMEGS) Clinical Practice Guidelines [18].

Although there are many different types of source localization algorithms (e.g., beamforming and current density methods), the ECD model forms the basis of most of these, and the single ECD model is the most well-studied and clinically validated MSI method [19]. Interictal epileptiform activity was first recognized on MEG in 1982 [20], and the ECD was successfully correlated to epileptiform activity on electrocorticography (ECoG) in 1994 [21]. Since then, there has been an abundance of literature illustrating the accuracy of the ECD model in localizing epileptogenic brain regions, as detailed in the subsequent sections. Then, in 2020, Bagić et al. outlined 10 common evidence-supported indications for MEG in epilepsy surgery [22]. These clinical scenarios are detailed in Table 2.

## 3. Stereotactic Electroencephalography (SEEG)

Stereotactic EEG was conceived by Jean Talairach in the Hospital Sainte-Anne, Paris, in the late 1950s [23,24]. The technique was then refined primarily within a single center until the late 20th century [23]. With the introduction of new technology, such as robotic assistance, SEEG was established within the Cleveland Clinic Epilepsy Center and then rapidly expanded throughout the United States (US) [23,25]. In fact, since 2013, there has been substantial growth in the use of SEEG in the US at National Association of Epilepsy Centers (NAEC) [25].

Interestingly, and perhaps for a variety of practical reasons (e.g., cost, technical expertise, etc.), MEG has not experienced similar growth [9,26,27]. In higher-volume NAEC, MEG is more frequently incorporated into epilepsy presurgical evaluation [27]. However, overall, MEG was only utilized in a median of 5% of surgical evaluations [9]. The dearth of MEG in epilepsy presurgical evaluations has been recognized by other authors who have stated, “…MEG remains underutilized in many epilepsy centers…Our patients with drug-resistant epilepsy deserve nothing less than our most sophisticated diagnostic approaches” [28].

Although there has been significant growth in the utilization of SEEG, the basic principles of this technique have remained largely unchanged since the first SEEG implantation on 3 May 1957. Specifically, a successful SEEG investigation requires a detailed understanding of the patient’s seizure semiology [23,24,29,30,31,32,33,34].

An SEEG evaluation provides several advantages over a subdural electrode (SDE) study. First, SEEG is associated with fewer complications compared to an SDE study [35]. Secondly, compared to SDEs that often require a craniotomy for implantation, SEEG tends to be better tolerated by patients [23,35]. Finally, deep cortical and subcortical structures, e.g., the mesial temporal lobe, cingulate cortex, thalamus, etc., as well as bilateral epileptogenic sources, can be explored more effectively with SEEG [24,35,36,37,38,39].

A common critique and potential limitation of SEEG is a paucity of spatial sampling [35,40]. However, this criticism may overlook and underappreciate the SEEG methodology. Stereotactic EEG should not be considered a depth electrode approach to identifying a seizure focus. Instead, SEEG is three-dimensional investigation of cerebral networks based on anatomo-electroclinical correlation. Therefore, the spatial sampling is directed specifically within suspected seizure onset and propagated brain regions.

Seizure semiology remains the cornerstone for developing an SEEG implantation strategy. However, as the following sections illustrate, MEG can augment or serve as an important partner to seizure semiology and create a stronger hypothesis for an SEEG investigation.

## 4. MEG Augmentation of SEEG Evaluation

### 4.1. Seizure Semiology Overview

A full assessment of seizure semiology is beyond the scope of this review. However, a brief overview of seizure semiology is essential as it remains the foundation for a successful SEEG evaluation [23,24,29,30,32,33,34]. Specifically, the symptoms and signs experienced during an epileptic seizure potentially illuminate the spatial and temporal spread within cortical and subcortical networks. As such, these clinical features provide insight into brain regions in which epilepsy seizures originate and propagate.

The two most common focal epilepsies in adults are temporal lobe epilepsy and frontal lobe epilepsy [33,41,42,43]. The clinical features within these types of epilepsy, particularly frontal lobe epilepsy, can vary significantly, and to better understand the semiology, both temporal lobe epilepsy and frontal lobe epilepsy can be divided into subtypes [44,45,46,47]. For example, people with the mesial temporal subtype often have a well-described and reproducible history with a febrile seizure or an insult early in life, e.g., central nervous system infection [42]. The semiology also has similar features characterized by abdominal or experiential auras, impaired consciousness, and automatisms [42,44]. Conversely, a lateral temporal subtype may present with an auditory aura. In these seizures, automatisms are less common, but focal-to-bilateral tonic–clonic (FBTC) seizures may occur regularly [44].

Frontal lobe seizures display a wide range of clinical features that can, to some degree, be organized within particular regions [43,46,47,48]. However, reciprocal connections within frontal lobe networks may produce similar clinical features within different brain regions. Therefore, a minimal understanding of the diversity of frontal lobe seizures is necessary for a successful SEEG study. For example, seizures arising from the precentral cortex often present with distal-to-proximal rhythmic clonic activity (e.g., Jacksonian March), while seizures emanating from the prefrontal cortex may exhibit a complicated semiology with non-integrated/disjointed hyperkinetic movements [47,48].

Parietal lobe epilepsy and occipital lobe epilepsy comprise approximately 20% of focal epilepsy cases [49,50]. Although the clinical semiology can be variable in these focal epilepsies, contralateral somatosensory symptoms are a common feature in PLE [50]. Seizures arising from the visual cortex may present with visual hallucinations [50]. For example, elementary visual hallucinations, e.g., multicolored objects such as a circle or square within a visual field, suggest a seizure emanating from the primary visual cortex [50]. If the subsequent clinical features are speech impairments and automatisms, this could represent an occipital seizure propagating to the temporal lobe, possibly via the inferior longitudinal fasciculus.

Across all these focal epilepsies, the semiology serves as the cornerstone for a successful SEEG evaluation. That is, the implantation strategy is unique to each patient, and not only is a temporal lobe epilepsy implantation different from a frontal lobe epilepsy implantation, but an SEEG implantation for a mesial temporal subtype evaluation will also differ from that for a lateral temporal subtype.

### 4.2. Illustrative Cases

Two case reports are presented to demonstrate the additive value that MEG provides to seizure semiology in an SEEG investigation. These two cases depict the two most common types of focal epilepsy: temporal lobe epilepsy (Case 1) and frontal lobe epilepsy (Case 2). The cases also illustrate two of the 10 common evidence-supported indications for MEG in epilepsy surgery [22]. Specifically, Case 1 corresponds to MEG indication #2 (MRI-negative with suspected mesial temporal onsets), while Case 2 meets the criteria for indication #5 (Diagnostic or therapeutic re-operation). Finally, other clinical MEG features such as ictal MEG (Case 1) and MRI re-review (Case 2) will be outlined to further demonstrate the necessity of MEG in SEEG evaluations.

#### 4.2.1. Case 1

The patient is a 35-year-old right-handed female with a history of drug-resistant focal epilepsy manifesting as focal impaired consciousness (FIC) seizures and focal-to-bilateral tonic–clonic (FBTC) seizures. There is a remote family history of seizures. Otherwise, there are no obvious epilepsy risk factors. Her seizures began around the age of 10 years and have continued despite ASM trials including carbamazepine, phenytoin, levetiracetam, clonazepam, lacosamide, and brivaracetam. At the time of her epilepsy presurgical evaluation, her ASM regimen consisted of 800 mg lamotrigine daily and 40 mg clobazam at bedtime.

Her seizure semiology is as follows: olfactory or gustatory hallucination followed by paresthesia in her face, dizziness, tachycardia, and then a loss of consciousness. After this, there are bimanual automatisms and dystonic posturing of the left hand. In addition, left rhythmic ictal non-clonic hand movement (RINCH) has been witnessed. Her brain MRI was non-lesional. A fluorodeoxyglucose positron emission tomography (FDG-PET) study was normal. Neuropsychology revealed normal cognition and was otherwise non-lateralizing. Although her FBTC seizures were well-controlled, she continued to have between one and three FIC seizures per month. Her scalp EEG, brain MRI, and FDG-PET scan are shown in Figure S1A–D.

A total of 122 min of simultaneous scalp EEG and MEG was obtained on RICOH MEG in epochs up to 15 min in length using the following parameters: DC coupled instead of high-pass filter, a low-pass filter of 1000 Hz, and a sampling rate of 2000 Hz. Waveforms were visually inspected with a bandpass of 0.1–500 Hz with a notch filter. Scalp EEG was reviewed using Nihon Kohden (https://www.nihonkohden.com/index.html, access date on 19 July 2026) and RICOH MEG software (https://www.idsa.org/awards-recognition/idea/idea-gallery/ricoh-meg/, access date on 19 July 2026). Selected spikes in the MEG were analyzed using the equivalent current dipole (ECD). A single dipole was selected to represent each sharp wave and/or spike-and-wave discharge. The prespecified statistical thresholds for dipole selection in this MEG center included a goodness of fit greater than 85%, correlation greater than 90%, dipole moment between 100 and 500 nAm, and a confidence interval ≤ 5 cm^3^. In general, the ECD was selected from the onset, or near-onset, of the epileptiform discharge up to the point of maximum amplitude. The ECD calculation was performed using at least 35–45 axial gradiometers, which were chosen to best represent the contour plot of the magnetic flux.

**Figure 1:**
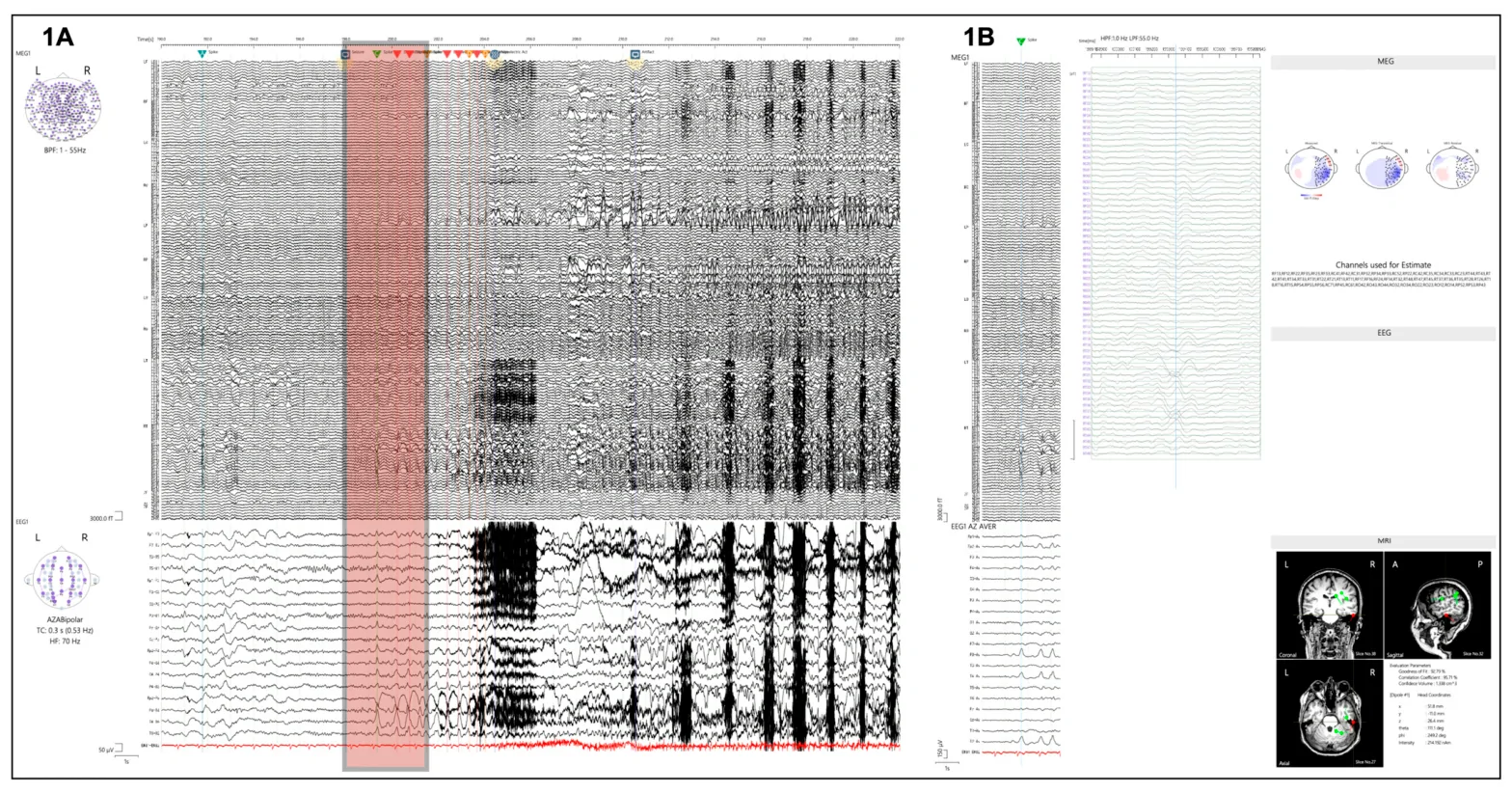
(**A**,**B**) Case 1—Seizure during MEG. Figure 1 illustrates a seizure recorded on RICOH MEG. In (**A**), the scalp EEG is arranged in an AP bipolar montage and the MEG represents the recording of 160 axial gradiometers arranged in the following order: LF—left frontal, RF—right frontal, LC—left central, RC—right central, LP—left parietal, RP—right parietal, LO—left occipital, RO—right occipital, LT—left temporal, RT—right temporal, ZF—midline frontal, ZC—midline center, and ZP—midline parietal. The red rectangle in (**A**) represents the area analyzed by the ECD for the initial ictal spike-wave discharge on MEG. (**B**) shows 1 s of the whole-head MEG and EEG (average montage) and then a 1 s epoch of the ECD analysis with the contour map (top right) and ECD localization (bottom right). Note: This ECD mapped with lobar localization is consistent with previously published ictal MEG studies [51,52,53].

**Figure 2:**
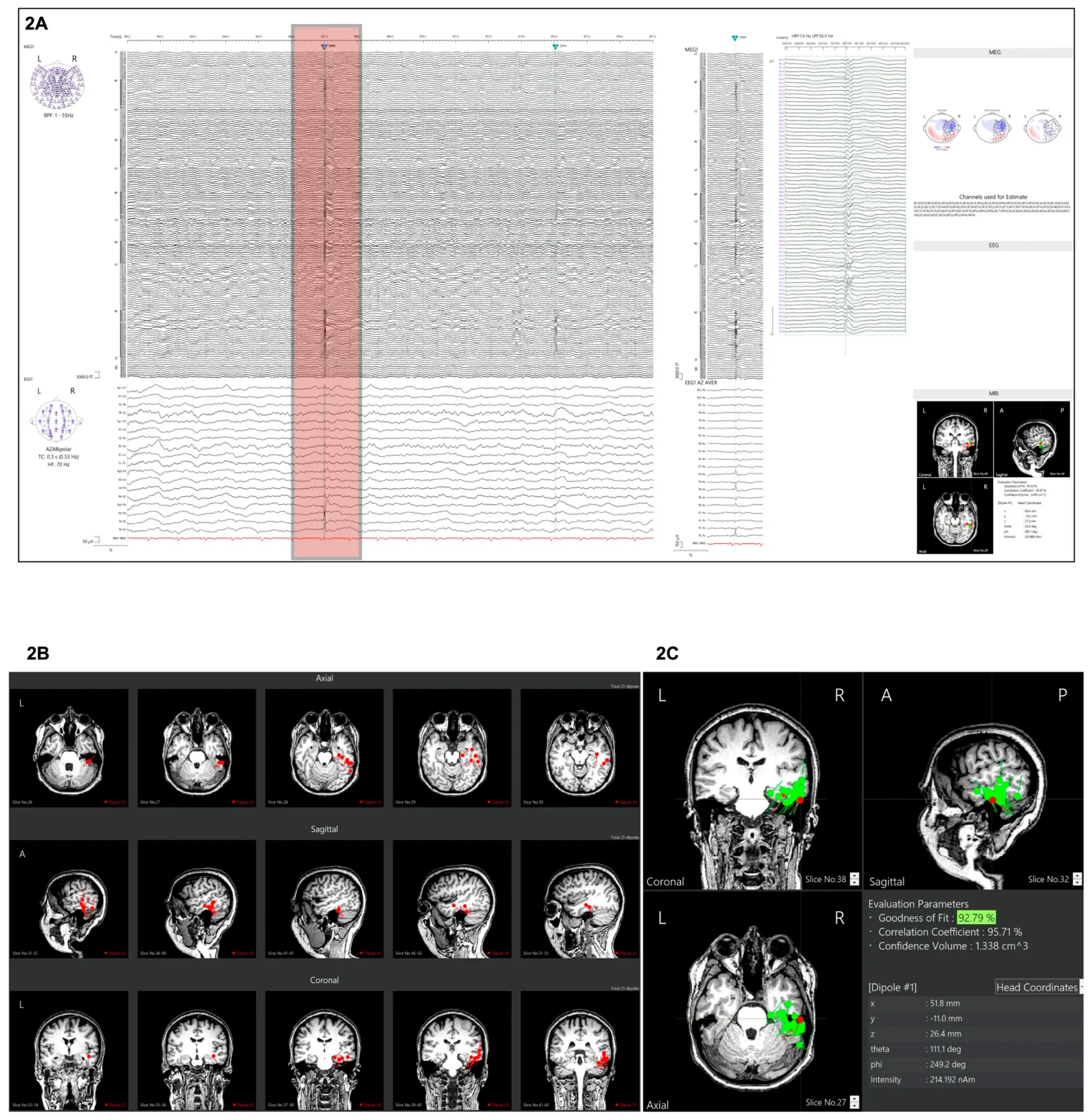
(**A**–**C**) Case 1—MEG interictal and ictal ECD. (**A**) is a representative example of a right temporal interictal epileptiform discharge on EEG and MEG (delineated by the red rectangle). In the right panel, there is a 1 s epoch of the interictal epileptiform discharge (EEG in an average montage) with the upper right panel demonstrating the region of interest on MEG and the counter map. The ECD localization is in the bottom panel of (**A**). (**B**) is an example of all 21 interictal ECDs, while (**C**) shows the relationship between the ictal ECD (red ECD in crosshairs) and the interictal ECD. Note: Green ECDs are not in the same plane as the red ECD. The ictal ECD is illustrated by a red ECD within the crosshairs. Based on her semiology and these MEG findings, an SEEG hypothesis was derived, detailed in Figure 3A–D.

During the study, one seizure was recorded and a total of 21 interictal epileptiform discharges met ECD selection criteria. The ECD of the initial MEG spike-wave during the seizure is shown in Figure 1. Figure 2A illustrates an interictal epileptiform discharge analyzed with the ECD, while Figure 2B reveals all analyzed interictal and ictal epileptiform discharges and 2C compares the ictal ECD to the interictal epileptiform discharges. The ECD of the initial MEG spike-wave in the seizure was localized to the right inferior lateral temporal lobe, while the interictal ECD mapped throughout the right temporal lobe in a fairly tight cluster with relatively consistent dipole orientation. These findings are supportive of an epileptic source within the right temporal lobe.

**Figure 3:**
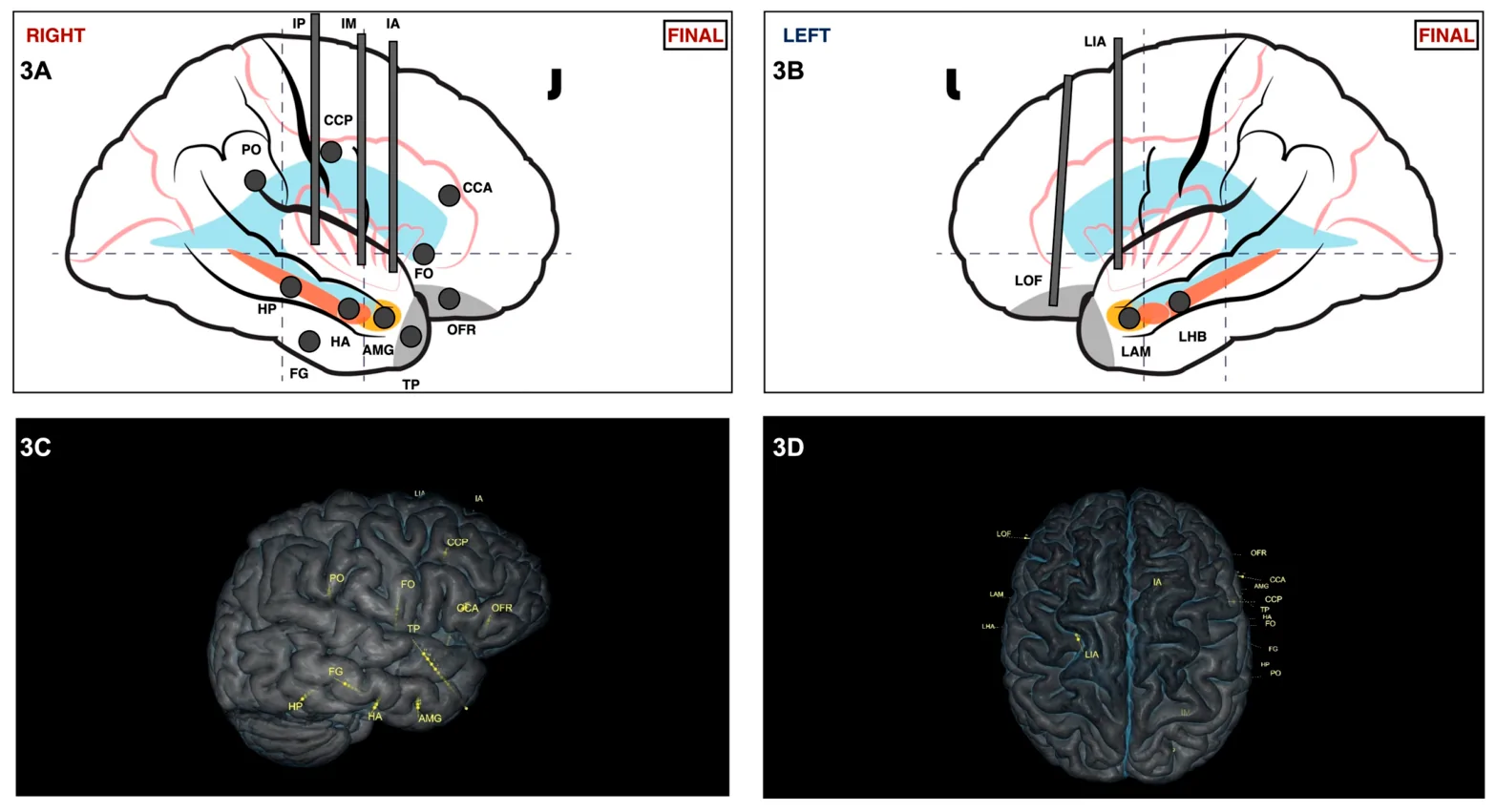
(**A**–**D**) Case 1—SEEG implantation. Figure 3 outlines the SEEG implantation strategy ((**A**), right brain; (**B**), left brain). Gray circles denote an orthogonal SEEG implantation, while the vertical gray rectangles represent vertical and oblique SEEG positioning. Abbreviations are based on internal, program-specific nomenclature and are as follows: OFR—orbitofrontal region, FO—frontal operculum, CCA—anterior cingulate cortex, CCP—posterior cingulate cortex, PO—parietal operculum, IA—anterior insula, IM—mid insula, IP—posterior insula, TP—temporal pole, AMG—amygdala, HA—anterior hippocampus, HP—posterior hippocampus, FG—fusiform gyrus, LAM—left amygdala, LHB—left hippocampal body, LIA—left anterior insula, and LOF—left orbitofrontal region. (**C**,**D**) are three-dimensional reconstructions of the SEEG electrode implantation in the right sagittal view and axial orientation.

**Figure 4:**
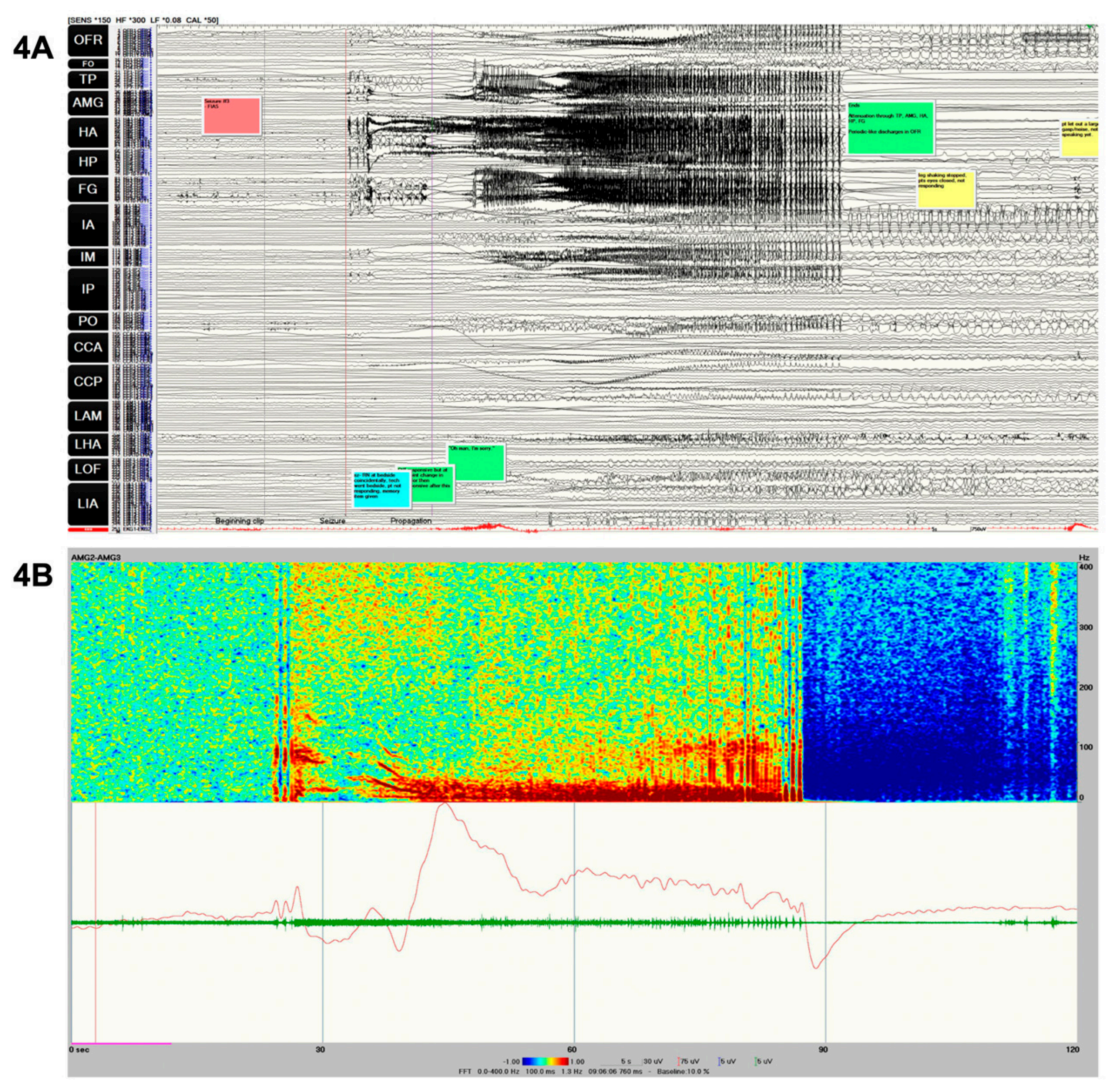
(**A**,**B**) Case 1—SEEG findings. (**A**) is SEEG data shown in a 2 min epoch with a wide bandpass of 0.08–300 Hz (sensitivity 150 µV; sampling at 1000 Hz). (**B**) is a Wavelet analysis (proprietary software within Nihon Kohden) of the ictal activity at AMG electrode contacts 1–2. The findings in (**B**) are similar to the ictal fingerprint described in Grinenko et al.’s study [54] and support a seizure onset within the right mesial temporal lobe. Before SEEG implantation, a seizure onset within the right mesial temporal lobe was suspected based on the patient’s seizure semiology. In addition, a seizure recorded during MEG localized an initial MEG spike-wave within the right basal temporal lobe. This lobar localization is consistent with previously published studies [51,52,53,55]. In this case, both ictal and interictal MEG enhanced the semiology-derived hypothesis for SEEG implantation, reinforcing several other studies [22,52,55,56,57,58].

Based on her semiology and MEG findings, an SEEG hypothesis was developed proposing seizure onset within the right temporal lobe with propagation through the right insula and then right frontal lobe. She then underwent an SEEG evaluation with asymmetric bilateral temporal coverage (there was a remote history of possible left temporal seizures) and right insula and right frontal lobe coverage (Figure 3A–D). During SEEG, several habitual seizures were recorded, with all seizures arising from the right temporal lobe (Figure 4A,B). Electrical stimulation mapping (ESM) reproduced habitual seizures arising from the right mesial temporal lobe. She then underwent a right anterior temporal lobectomy (ATL) and histopathology revealed findings most suggestive of focal cortical dysplasia (FCD) Type IIa. She has remained seizure-free since the right ATL (9 months at the last follow-up).

#### 4.2.2. Case 2

The patient is a 42-year-old right-handed female with a history of drug-resistant focal epilepsy manifesting as FIC and FBTC seizures. She has a remote history of traumatic brain injury (TBI) at the age of 7 years old. Her seizures began approximately 1.5–2 years after the TBI and have remained refractory to multiple ASMs, including phenobarbital, carbamazepine, phenytoin, levetiracetam, clobazam, and lamotrigine. At the time of her epilepsy presurgical evaluation, her ASM regimen consisted of oxcarbazepine 900 mg daily.

She completed an epilepsy presurgical evaluation at an outside institution that did not include MEG. She then completed intracranial EEG (iEEG) with SEEG and underwent a left frontal corticectomy. Unfortunately, within 12 months her seizures recurred and exhibited a similar semiology to that of her presurgical seizures. At the time of her evaluation in our center, she would have FIC seizures three days per week, sometimes with four seizures in one day.

**Figure 5:**
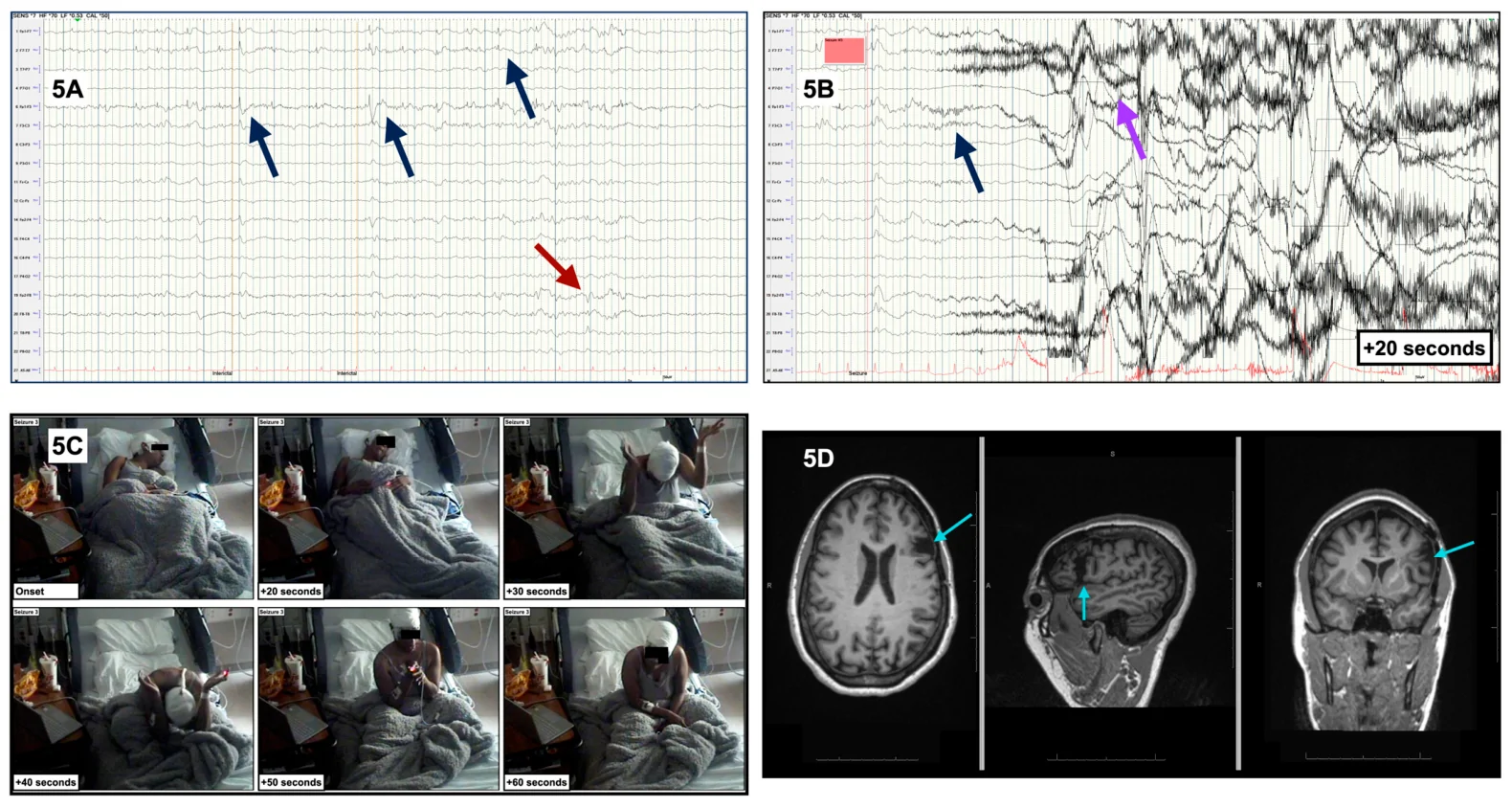
(**A**–**D**) Case 2—Scalp EEG findings, semiology, and postoperative brain MRI. (**A**) is an EEG reviewed in an AP bipolar montage (sensitivity 7 µV, high-frequency filter 70 Hz, low-frequency filter 0.53 Hz) revealing interictal epileptiform activity within the left frontal region (dark blue arrows) and right temporal region (red arrows). (**B**) is the third seizure recorded during video-EEG monitoring on scalp EEG in an AP bipolar montage (sensitivity 7 µV, high-frequency filter 70 Hz, low-frequency filter 0.53 Hz). There is lower-amplitude, rhythmic beta-range activity (dark blue arrow) within the left fronto-central region. After this, the EEG is largely obscured by a myogenic artifact (purple arrow). In (**C**), screenshots of the ictal semiology are depicted. At +20 s, there is an arousal from sleep. By +30 s there are gestural movements of the upper extremities followed by flexion at the waist (+40 s), clasping of both hands at +50 s, and behavioral arrest at +60 s. These features are suggestive of frontal lobe seizures and specifically seizures arising from the prefrontal cortex [33,43,45,46,47]. (**D**) is a postoperative brain MRI obtained after the initial resective surgery completed at an outside institution. Note: An MEG was not obtained in the initial epilepsy presurgical evaluation.

Her habitual FIC seizure is characterized as follows: She arouses from sleep, at which time she sits upright and flexes at the waist. During this time, there is a facial grimace and gestural movements of her bilateral upper extremities consisting of repetitive clapping and then clasping of both her hands, and she is unresponsive. Representative scalp EEG, semiology, and brain MRI are provided in Figure 5A–D. Her neuropsychology evaluation revealed deficits in executive functioning (suggestive of frontal lobe dysfunction). However, the testing profile was non-lateralizing.

**Figure 6:**
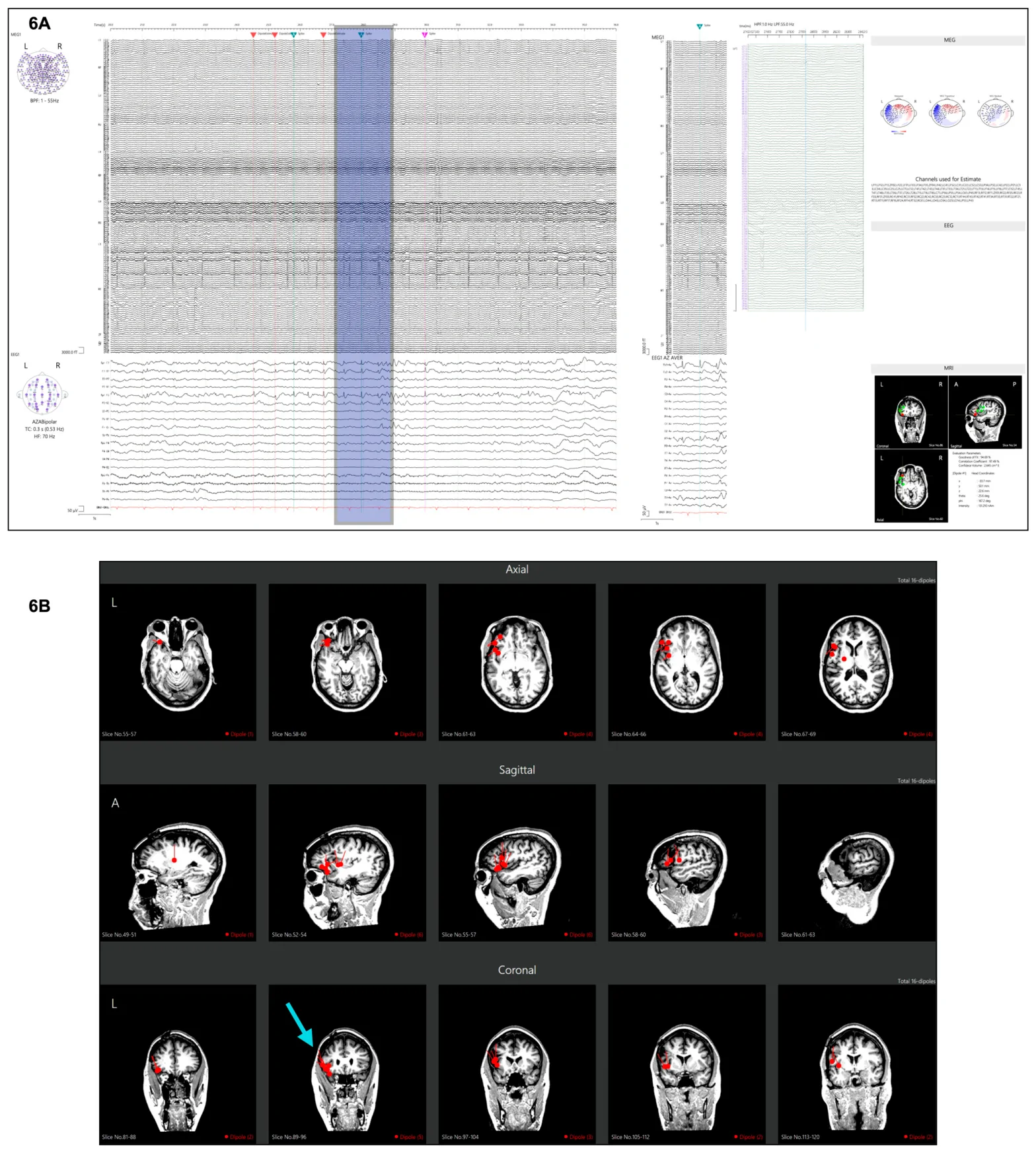

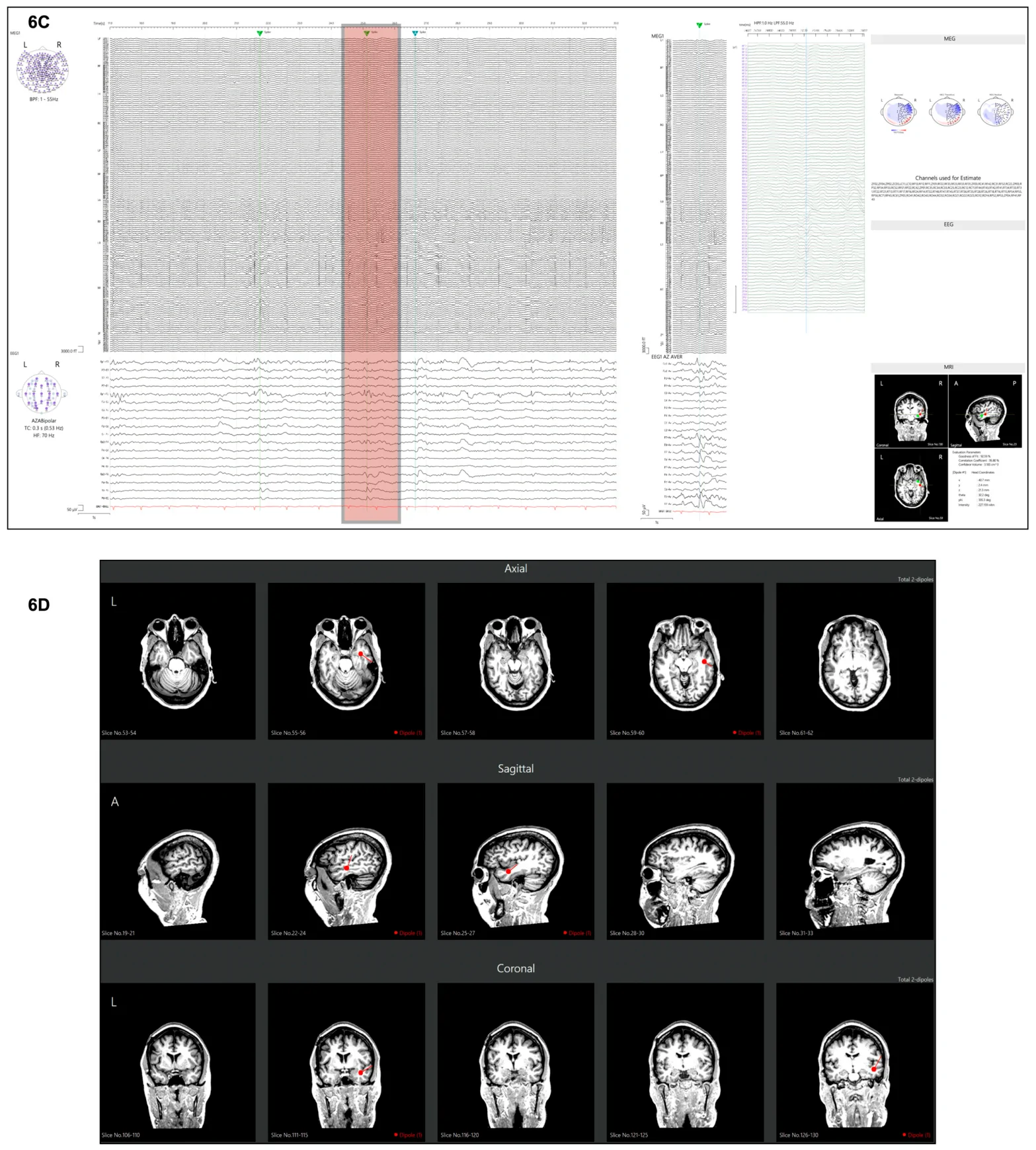
(**A**–**D**) Case 2—MEG interictal epileptiform activity. (**A**) is simultaneous scalp EEG and MEG. The blue rectangle is a 1 s sample selected for ECD analysis of a left hemisphere interictal epileptiform discharge on EEG and MEG. The right panel shows a 1 s epoch of EEG (average montage) and MEG. The region of interest in MEG is represented with the corresponding dipolar map to the right of the interictal epileptiform discharge. In the bottom right panel of (**A**) is the interictal ECD localization co-registered to the patient’s brain MRI. (**B**) is the cluster of left hemisphere ECD mapping to the left anterior frontal lobe, with several ECD mappings to the left orbitofrontal region (light blue arrow). This cluster of ECD prompted a brain MRI re-review that demonstrated an irregular gyrus and sulcus suggestive of an FCD. Magnetoencephalography-guided MRI re-review has repeatedly shown identification of previously underrecognized cortical malformations [59,60,61,62,63]. (**C**) illustrates a right temporal interictal epileptiform discharge in MEG and EEG. In the adjacent panel, the interictal sharp-and-wave discharge mapped to the right temporal lobe (adjacent panel). Finally, (**D**) illustrates the location of 2 right temporal sharp-and-wave discharge ECDs. Although scalp EEG revealed findings suggesting independent right temporal interictal epileptiform activity, the findings in the left hemisphere on MEG were more prominent. The presence of additional, unique MEG information, e.g., “diagnostic gain,” has been reported in several MEG studies and further informs SEEG implantation [54,64,65]. These findings supplemented the SEEG implantation strategy for this patient. However, the left frontal lobe ECD findings proved to accurately identify the seizure onset zone (Figure 8 and Figure 9, detailed below).

**Figure 7:**
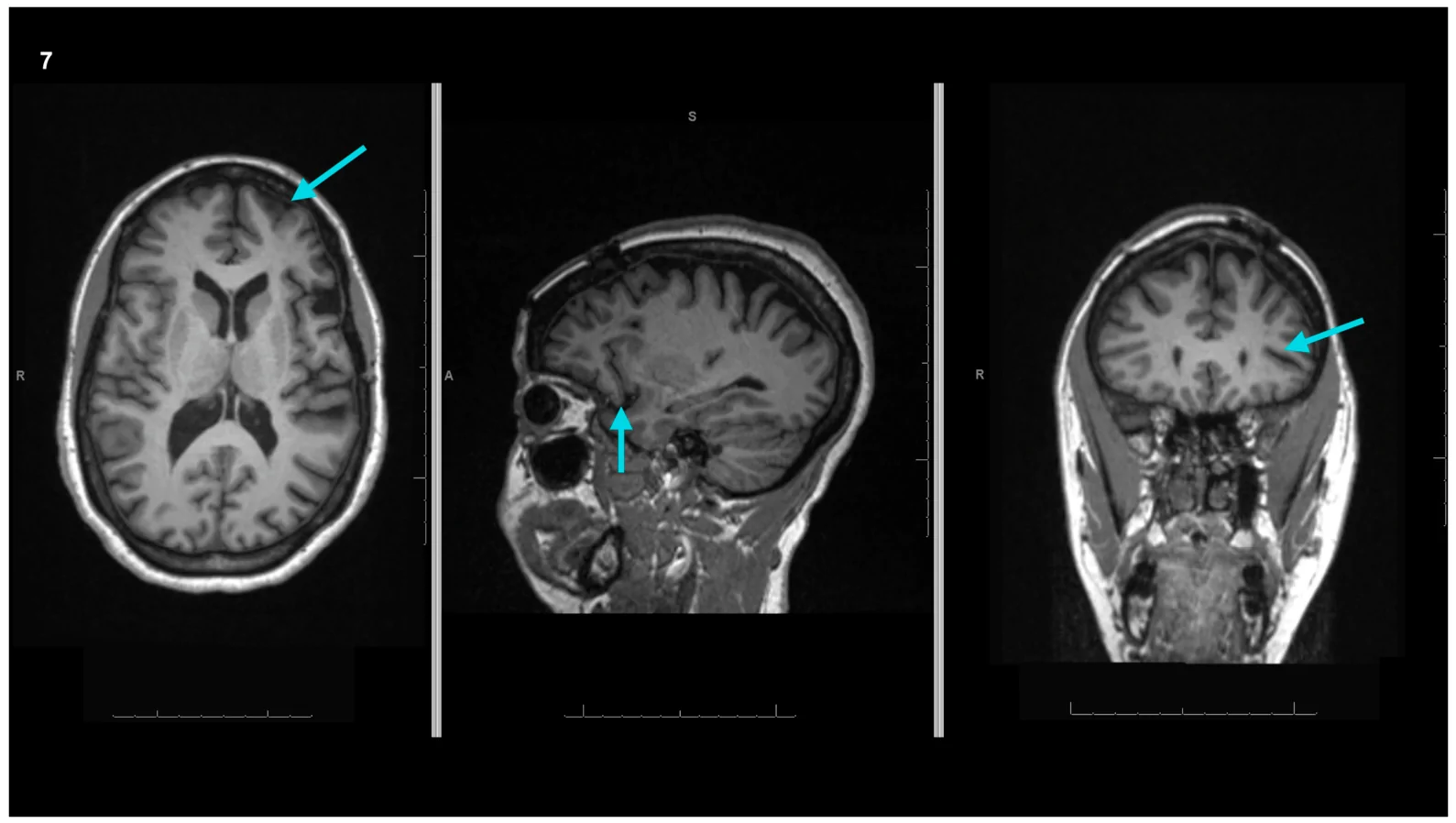
Case 2—Brain MRI re-review. After the MEG was presented in the patient management conference, the patient’s brain MRI was re-examined. The light blue arrow points to an abnormal gyrus and sulcus within the left orbitofrontal lobe (Magnetization-Prepared RApid Gradient Echo, MP-RAGE, sequences). These findings reinforce the importance of brain MRI revaluation after an MEG displays a cluster of ECDs within a specific brain region [60,61,63].

She then completed 127 min of simultaneous scalp EEG and MEG with the same protocol as Case 1. A total of 18 spike-waves met ECD inclusion criteria (16/18 mapping to the left frontal lobe and 2/18 localizing to the right temporal lobe) and examples of the interictal ECD are shown in Figure 6A–D. Based on the left frontal lobe ECD, the brain MRI was re-reviewed and an atypical gyrus and sulcus were identified within the left orbitofrontal region (Figure 7).

**Figure 8:**
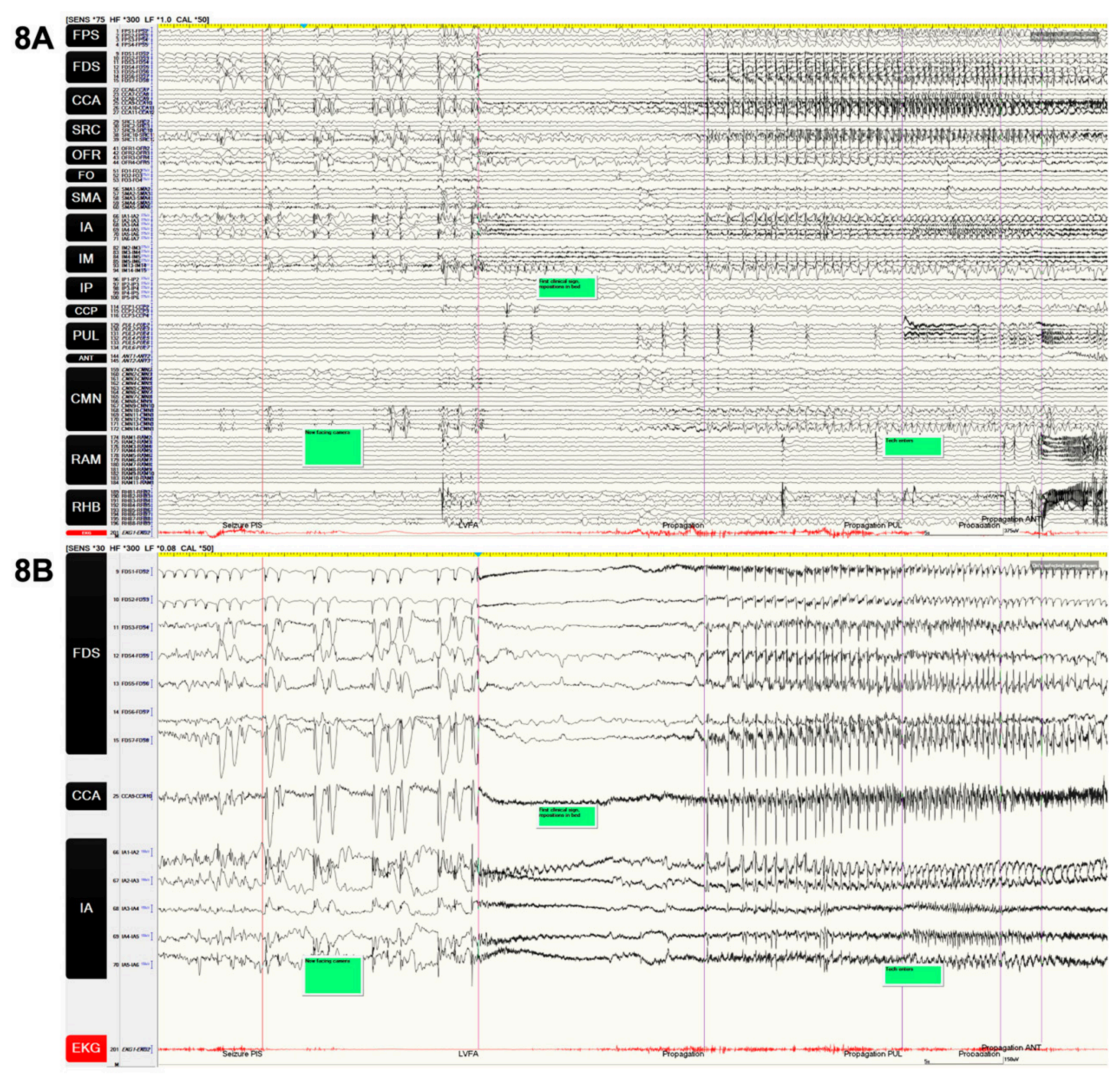
(**A**,**B**) Case 2—SEEG findings. (**A**,**B**) reveal findings from a spontaneous habitual seizure recorded during an SEEG investigation. The depth electrodes are labeled as follows: FPS—frontal pole superior FDS—frontal dysplastic sulcus, CCA—cingulate cortex anterior, SRC—superior resection cavity, OFR—orbitofrontal region, FO—frontal operculum, SMA—supplementary motor area, IA—anterior insula, IM—middle insula, IP—posterior insula, CCP—posterior cingulate cortex, PUL—pulvinar with superficial contact within the left superior temporal gyrus, ANT—anterior nucleus of the thalamus, CMN—centromedian nucleus, RAM—right amygdala, and RHB—right hippocampal body. (**A**) is a 60 s epoch of habitual seizure #3 (sensitivity 75 µV, high-frequency filter 300 Hz, low-frequency filter 1 Hz). Note: Preictal spiking (PIS) occurs in contacts FDS, CCA, SRC, OFR, IA, and IM, followed by attenuation in FDS, CCA, and IA. (**B**) is a 60 s epoch in the region of interest with a wide bandpass (sensitivity 30 µV, high-frequency filter 300 Hz, low-frequency filter 0.08 Hz) further illustrating the attenuation and over-riding low-voltage faster activity (LVFA).

**Figure 9:**
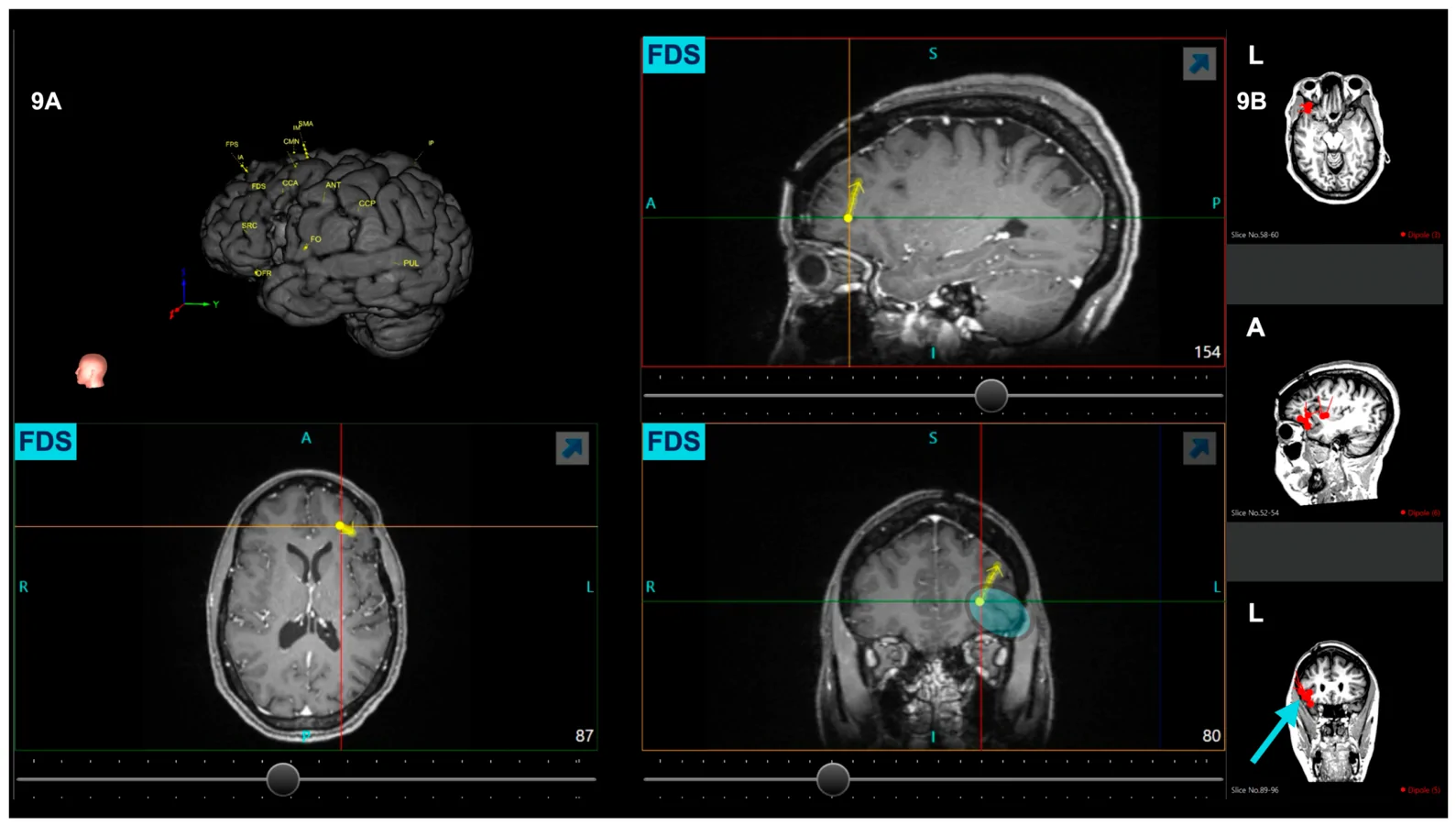
(**A**,**B**) SEEG and MEG findings. (**A**) is the three-dimensional reconstruction of the SEEG implantation (upper left panel) and co-registration of the SEEG depth electrode within the patient’s brain MRI (yellow arrow; FDS—frontal dysplastic sulcus). The light blue oval outlines the irregular gyrus and sulcus within the left orbitofrontal region that several MEG ECDs mapped to and from which spontaneous seizures emanated on SEEG. (**B**) is the cluster of MEG ECD mappings to the abnormal gyrus and sulcus in the left orbitofrontal region (light blue arrow). The findings in this study reinforce the published literature insofar as MEG ECD can accurately identify epileptogenic brain regions [21,22,54,55,57,58,59,62,63,66,67], identify previously underappreciated cortical malformations [60,61,63], accurately localize a seizure onset zone [64], and augment the semiology and SEEG implantation strategy [22,55,57,68].

Based on her semiology and MEG findings, an SEEG hypothesis was developed proposing seizure onset within the left orbitofrontal region, specifically the region of the MEG ECD and atypical gyrus on structural brain MRI. After seizure onset, propagation would include structures within the left frontal lobe, left insula, and right temporal region. During SEEG, numerous seizures were recorded arising from the contacts FDS (frontal dysplastic sulcus), as detailed in Figure 9A,B. A final epilepsy surgery had not been completed at the time of this report.

### 4.3. MEG Informing and Predicting Epileptic Activity on SEEG

The value of MEG in epilepsy presurgical evaluation has been repeatedly demonstrated over the last 30 years, beginning with Nakasato et al.’s 1994 [21] publication illustrating “excellent agreement between MEG and ECoG as well as surgical outcome when the area identified as the focus was included in the excision.”. Subsequent publications reinforced the utility of MEG in the epilepsy presurgical evaluation. In 2008, Sutherling et al. noted that MEG provided information that changed treatment in approximately 9% of patients and provided non-redundant information in 1/3 of patients [56]. In a more recent review, Geller et al. estimated there is a “diagnostic gain” from MEG in 16.8–18% of cases [69]. Perhaps in one of the most impactful MEG studies, Knowlton et al. reported that MSI localized the seizure onset zone (SOZ) with nearly the same frequency as iEEG [64].

In case series from Erlangen, Germany, published in 2003 and 2005, the accuracy of MEG in identifying epileptogenic brain regions was demonstrated [66,70]. There were also favorable surgical outcomes when MEG findings were included in the resection [66,70]. These findings were reinforced in a series of 1000 MEG studies, which reported that complete resection of the MEG findings was associated with a significantly higher probability of seizure-freedom after surgery [71].

The utility of MEG in epilepsy presurgical evaluations was also illustrated in case series from the Cleveland Clinic Epilepsy Center in 2013 and 2014 [59,67]. In each of these studies, complete resection of an MEG cluster was associated with a higher chance of postoperative seizure-freedom [59,67]. In addition, Almubarak et al. noted anatomical concordance between MEG and iEEG, and complete resection of the MEG focus increased the likelihood for postoperative seizure-freedom [59].

In 2015, Englot et al. evaluated 132 patients who underwent epilepsy surgery after completing an MEG. A total of 85% of patients with concordant and specific MEG findings (no ECD in other lobes) became seizure-free [60]. Conversely, only 37% of patients with discordant or nonspecific MEG findings (>10% of ECD in distal brain regions) became seizure-free [60]. In addition, the authors reported that MEG results promoted a re-review of brain MRI and, in some cases, identified previously unrecognized focal cortical dysplasia (FCD) [60]. These findings also portended a higher likelihood of postoperative seizure freedom [60].

Although a review of the literature regarding the ability of MEG to identify previously underappreciated or unrecognized cortical malformations is beyond the scope of this paper, there is a substantial number of publications dating back to 2002 detailing this important finding [61,62]. Some authors have suggested that MSI can be used to find FCDs in MRI-negative epilepsy patients [62,63,72]. Additionally, other authors have revealed the relationship between the MRI lesion (FCD), MSI, and iEEG (SEEG), and reported that modeling the MEG spike-waves can localize the SOZ in patients with FCD [68].

Taken together, these studies have repeatedly illustrated the utility of MEG in epilepsy presurgical evaluations. However, many of these studies did not specifically address the impact of MEG on the SEEG evaluation.

One of the first and largest case series addressing the importance of MEG in an SEEG evaluation was from the Cleveland Clinic Epilepsy Center [57]. This study evaluated 50 consecutive patients who underwent MEG and then SEEG [57]. Consistent with previous case reports and case series [59,60,67], complete resection of MEG clusters resulted in a higher chance of seizure-freedom compared to limited and no-resection groups [57]. Moreover, and perhaps as importantly, patients had a significantly higher chance of seizure-freedom when SEEG completely sampled the area identified by MEG as opposed to incomplete or no sampling of the MEG findings [57]. These findings suggest that MEG identifies epileptogenic brain regions that must be sampled with SEEG to better understand and delineate the epileptogenic zone network (EZN).

More recently, similar findings were reported in a case series of 42 patients in the Shanghai Ruijin Hospital [58]. In this case series, postoperative outcomes were improved when MEG was concordant with SEEG [58]. However, again, outcomes were also improved when the MEG findings were completely sampled by SEEG [58].

Beyond the MEG cluster, the ECD characteristics, e.g., dipole orientation and statistical parameters (Q, CV, chi-square, etc.; see Table 1) also inform findings on SEEG and surgical outcomes. In a case series of 40 patients with a positive MEG who subsequently underwent an SEEG evaluation at the Baylor College of Medicine, the CV was a significant predictor of epileptic activity on SEEG [55]. Overall, this study demonstrated the ability of MEG to predict ictal activity on SEEG [55]. Specifically, 14–17 ECDs within a cluster had 75.9–82.5% sensitivity for detecting ictal activity on SEEG. Each additional ECD assigned to a cluster increased the likelihood of ictal activity on SEEG by 9% [55].

Murakami et al. also reported that patients with ECD clusters that exhibited a stable orientation perpendicular to the nearest major sulcus had a better chance of seizure-freedom compared to other ECD orientations [57]. Laohathai et al. also noted the importance of ECD orientation in a comprehensive review of clinical MEG interpretation in epilepsy [13]. For example, ECD in the temporal lobe may be divided into three groups: (1) anterior temporal horizontal (ATH); (2) anterior temporal vertical (ATV); and (3) posterior temporal vertical (PTV) [13]. Outcomes of an ATL were improved when ATH and ATV ECDs were fully resected [13].

Magnetoencephalography is often considered an “interictal study.” That is, in most MEG studies, the analyzed epileptiform activity is interictal sharp and spike-and-wave discharges. However, seizures may be recorded in approximately 12% of MEG cases [51]. Not surprisingly, and consistent with MEG ECD localization of interictal epileptiform activity, ictal epileptiform activity on MEG can also localize epileptogenic brain regions [51,52,53,73].

In a case report from Zillgitt et al., a 42-year-old male experienced several seizures during a MEG study that exhibited MEG-unique preictal spiking [51]. The MEG preictal spiking correlated to the preictal spiking identified on SEEG [51]. The patient underwent responsive neurostimulation implantation and experienced a meaningful, approximately 90%, reduction in the frequency of disabling seizures [51].

Case series from the Cleveland Clinic Epilepsy Center reported similar findings [53,73]. In one case series, ictal MEG provided unique findings in 18% of patients and in those patients who underwent iEEG, there was sublobar concordance between MEG and iEEG in 75% of the cases [53]. These results appeared to be replicated in a large case series of 45 patients with seizure(s) during MEG [73]. Specifically, concordance between interictal and ictal ECD with iEEG was significantly associated with postoperative seizure-freedom [73].

### 4.4. Simultaneous MEG and SEEG

A concern, and sometimes criticism, of MEG is often related to the measured magnetic flux, which is often measured in the femtotesla (fT) range. When the strength of the electromagnetic source decreases by one over the radius squared, and the source is weak, MEG may become insensitive to deep cortical sources, such as those within the mesial temporal lobe [74]. These findings have been reinforced in several clinical studies [65,71,75].

For example, Leijten et al. reported that the yield of spikes in MEG for patients with mesial temporal lobe epilepsy (MTLE) is low [75]. In addition, ECD modeling only revealed partial correlation with electrocorticography [75]. Similar results were published by Wenneberg et al. in a small case series, where the authors that noted anterior temporal spike-waves did not localize to the hippocampus with MSI [65]. Finally, Rampp et al. reported the potential limited diagnostic value of MEG in patients with MTLE, insofar as the MEG results did not appear to significantly affect postsurgical outcomes [71].

However, other MEG studies have illustrated the ability of MSI to identify deep cortical sources within the perisylvian region [76], as well as mesial temporal structures [62,77]. With respect to MTLE, Kaiboriboon et al. demonstrated that MSI can identify spikes within the mesial temporal region [77]. The possible utility of MEG within temporal lobe epilepsy was included as one of the ten evidence-supported indications for clinical MEG, specifically in MRI-negative patients with a suspected mesial temporal SOZ [22].

Despite the many advantages SEEG has over SDE, the most significant limitation of SEEG is spatial sampling and sampling bias [35,78]. However, a strong semiology-driven hypothesis may overcome the limitation of spatial sampling [23,24,29,30,31,32,33,44,48]. In addition, as detailed above, MEG has demonstrated utility in identifying epileptogenic brain regions subsequently identified and delineated with SEEG [22,51,53,55,57,58,59,67,72,73]. The combination of these two modalities could potentially mitigate the limitations of both diagnostic modalities. That is, simultaneous MEG-SEEG can provide whole-head coverage and explore deeper brain regions [40,79,80,81,82].

One of the first case reports implementing simultaneous MEG-SEEG was published in 2016 [40]. Although habitual seizures recurred after surgery, the authors demonstrated the feasibility of simultaneous MEG-EEG [40]. The authors concluded that “Simultaneous recordings provide a global brain recording and overcome the inherent SEEG limited spatial sampling” [40].

In 2019, Pizzo et al. completed simultaneous MEG-SEEG in 14 patients [79]. Using an SEEG-triggered analysis, epileptiform activity localizing to the mesial temporal structures (amygdala and hippocampus) on MEG was identified in eight patients [79]. In another study of simultaneous MEG-SEEG in six patients with TLE, 33% of spikes localized to the hippocampus were also detected by MEG, albeit with lower amplitude and a higher signal-to-noise ratio [81]. For example, some mesial temporal spike-waves identified on MEG and analyzed with the ECD had a dipole moment (Q) between 26.1 and 61 nAm and a goodness of fit (GoF) between 68 and 89.6%, below some of the prespecified ECD thresholds considered acceptable in clinical practice [13,81].

Simultaneous MEG-SEEG not only supplements the potential limitations of each diagnostic modality but reinforces the ability of MEG to detect epileptiform activity also identified in SEEG evaluations and dispels some previously held MEG assertions. Specifically, whole-head MEG overcomes the sampling bias encountered in SEEG evaluations. In addition, simultaneous MEG-SEEG appears to have concordance in identifying epileptiform activity, and the resection of concordant MEG-SEEG epileptiform activity may lead to improved postoperative outcomes [80,81,82]. Finally, in some of these studies, MEG detected deep epileptiform activity, including within the mesial temporal lobe [79,80,81]. The latter finding may diminish the longstanding belief that MEG is insensitive to deep cortical sources.

## 5. Conclusions

Drug-resistant focal epilepsy is common and epilepsy surgery is a well-established treatment option for these patients. There are several diagnostic studies utilized in an epilepsy presurgical evaluation, but a detailed seizure semiology remains the cornerstone of a successful SEEG evaluation. Despite its persistent underutilization in epilepsy presurgical evaluations, there is an abundance of evidence illustrating the utility of MEG in the epilepsy presurgical evaluation. Furthermore, in SEEG evaluations, MEG has repeatedly demonstrated an ability to accurately identify intracranial epileptiform activity and delineate the EZN. As the utilization of SEEG continues to grow, seizure semiology and MEG should serve as the foundation for SEEG investigations.

## Figures and Tables

**Table 1:** Common ECD validation characteristics [13,15].

ECD Parameter	Definition
Dipole Moment (Q)	Strength of the dipole vector, measured in nanoamperes (nAm)
Correlation Coefficient (R)	Degree of association between the actual measured magnetic field data and the estimated forward solution data
Goodness of Fit (GoF)	How well the measured magnetic field agrees with the estimated field from an assumed dipole; 100% minus the normalized variance not explained
Root mean square (RMS)	Measure of the magnetic signal strength of the data at the latent for which the dipole is computed
Confidence volume (CV)	The spatial volume that encompasses the 95% probability of source localization
Chi-square test	Summation of normalized squared error into a single chi-square statistic

**Table 2:** The 10 common evidence-supported indications for MEG in epilepsy surgery [22].

1	Lacking or imprecise hypothesis regarding seizure onsets
2	MRI-negative with suspected mesial temporal onsets
3	Multiple lesions on brain MRI
4	Large lesions on brain MRI
5	Diagnostic or therapeutic re-operation
6	Ambiguous EEG findings suggestive of bilateral and/or generalized patterns
7	Intrasylvian suspect onset
8	Interhemispheric suspect onset
9	Insular suspect onset
10	EEG-negative, e.g., no EEG spikes

## Data Availability

The data presented in this study are available on request from the corresponding author. The data are not publicly available because of privacy and ethical restrictions.

## Supplementary Materials

The following supporting information can be downloaded at https://www.mdpi.com/article/10.3390/brainsci16080778/s1, Figure S1: Case 1—Noninvasive Diagnostic Studies; Figure S2: Clinical Evaluation of Interictal Epileptiform Activity Using the ECD, Step 1 in ECD Analysis; Figure S3: Steps 2–3 in ECD Analysis; Figure S4: Steps 4–5 in ECD Analysis.
