# Supplementary material for: Magnetoencephalography (MEG) Augments Semiology in Stereotactic Electroencephalography (SEEG) Evaluations
Source: Brain Sci. 2026 Jul 23;16(8):778. doi: 10.3390/brainsci16080778 (PMC13510708; doi:10.3390/brainsci16080778)
Supplement: Supplementary file 1 [file brainsci-16-00778-s001.zip › MEG-SEEG Suplementary Material Fig Descriptions.pdf]

Supplementary Material

Figure S1A–D: Case 1 – Noninvasive Diagnostic Studies

Figure 1A and 1B are scalp EEG of interictal and ictal epileptiform discharges. Figure 1A illustrates a right anterior temporal spike-and-wave discharge in a sphenoidal montage (Sensitivity 7 μV; High frequency filter 70 Hz, Low frequency filter 0.53 Hz), while Figure 1B shows the seizure onset with medium-to-high amplitude, rhythmic, 2-3 Hz, sharp wave discharges over the right temporal region (Anterior-Posterior (AP) bipolar montage, Sensitivity 7 μV; High frequency filter 70 Hz, Low frequency filter 0.53 Hz). Figure 1C is a T2 brain MRI sequence in the coronal orientation through the bilateral mesial temporal lobe. Finally, Figure 1D is the FDG-PET scan that does not demonstrate hypometabolism within the mesial temporal lobes. These findings can be common in patients with temporal lobe epilepsy and are an indication for MEG (Bagic AI F. M., 2020).

Figures S2–4: Clinical Evaluation of Interictal Epileptiform Activity Using the ECD

The patient is a 36-year-old right-handed male with a history of drug-resistant focal epilepsy manifesting as FIC and FBTC. At the age of 17 years old, he experienced a left intracerebral hemorrhage from an arteriovenous malformation (AVM). He is status post left-sided decompressive craniectomy. Shortly after this, he developed seizures that remained refractory to several ASM trials including phenytoin, levetiracetam, oxcarbazepine, zonisamide, and brivaracetam. At the time of his MEG, his ASM regimen consisted of lacosamide 200 mg twice daily and lamotrigine 200 mg twice daily. This history falls into indication #4 in the 10 Common Evidence-Supported Indications for MEG in Epilepsy Surgery (Large lesions on brain MRI) (Bagic AI F. M., 2020). The simultaneous EEG and MEG recording parameters are detailed in Case 1 vignette.

Figure S2: Step 1 in ECD Analysis

The first step in an ECD analysis is the visual inspection for epileptiform activity during simultaneous MEG-EEG recording. In this figure interictal epileptiform activity is labeled with either a green triangle with the letter “C” or blue triangle with the letter “A” (representing different MEG spike populations). A red triangle within the right of the 15-second epoch is an interictal epileptiform activity analysis. The light blue rectangle represents a 1-second epoch of interest for ECD analysis. Note: the EEG discharge appears within a breach rhythm, while the corresponding interictal epileptiform activity on MEG is more pronounced.

Figure S3A–B: Steps 2-3 in ECD Analysis

The selected EEG and MEG are then analyzed in a 1-second epoch. Comparisons of the onset and peak of the epileptiform discharge are analyzed. With this discharge, a point immediately before the peak was chosen for analysis (Figure S3B). In Figure S3B, an

enlarged 1-second of the MEG data is reviewed with the selected channels of interest, represented by small black circles within the contour maps. There is an obvious dipolar (black arrows) pattern in the measured counter map with the red color denoting the magnetic flux out of the head and the blue color denoting the influx of the magnetic field. The second contour map is the theoretical magnetic field, while the third contour plot is the subtraction of the measured and theoretical magnetic fields. This interictal epileptiform discharge displayed a robust dipolar pattern than was amenable to ECD analysis.

Figure S4A–B: Steps 4-5 in ECD Analysis

Figure S4A reveals the ECD solution coregistered to the patient's brain MRI. The statistical/evaluation parameters in the bottom right panel (outlined by a red square) meet the inclusion criteria outlined in the ACMEGS Clinical Practice Guidelines and Laohathai et al.'s report on the practical interpretation of MEG in epilepsy (Bagic AI & Committee, 2011; Laohathai C, 2021). The GoF is 96.11% with an R of 98.17% and CV of 0.096 cm^3. The Q (reported as intensity in the panel) is 522.450 nAm. Finally, replication of the ECD analysis with at least 5 epileptiform discharges is suggested to report a potential epileptogenic source (Bagic AI & Committee, 2011). In this study, a total of 23 ECD were included in the ECD analysis (Figure S4B).
